# Analytical interference by IgM paraprotein causing platform-dependent electrolyte discrepancies: a case-based laboratory investigation

**DOI:** 10.11613/BM.2026.030901

**Published:** 2026-08-10

**Authors:** Sheng Wang, Xiaoyong Duan, Yulin Zou, Jianxiong Gui

**Affiliations:** 1 The Third Clinical Medical College of China Three Gorges University, Gezhouba Central Hospital of Sinopharm, Yichang, Hubei, China; 2 The Eighth Hospital of Wuhan, Wuhan, Hubei, China

**Keywords:** paraprotein interference, pseudohypercalcemia, pseudohyponatremia, platform-dependent discrepancy, Waldenström macroglobulinemia

## Abstract

Accurate measurement of serum electrolytes and calcium is essential for clinical management; however, monoclonal immunoglobulins, particularly IgM paraproteins, may cause misleading analytical interference. We report the case of an 85-year-old man admitted for low back pain whose serum sample showed marked inter-platform discordance in sodium and total calcium results. The Hitachi platform reported a “hypercalcemic crisis” (total calcium 4.95 mmol/L) and severe hyponatremia (125 mmol/L), whereas the Beckman Coulter AU platform showed only mildly elevated total calcium (2.89 mmol/L) and milder hyponatremia (133 mmol/L). This corresponded to a 71.3% difference in total calcium, despite both platforms using the Arsenazo III method. Direct ion-selective electrode measurements excluded true hyponatremia and hypercalcemia. Dilution studies, polyethylene glycol precipitation, reaction-curve inspection, and simulation experiments confirmed that IgM-related precipitation and optical scattering were the underlying mechanisms. Specifically, platform-specific mixing dynamics determined the magnitude of interference. Recognition of biochemical-clinical discordance and targeted method comparison prompted hematological evaluation and led to a diagnosis of Waldenström macroglobulinemia. This case highlights that unexplained analytical discrepancies may delay recognition of underlying disease if analytical interference is not appropriately investigated.

## Graphical abstract

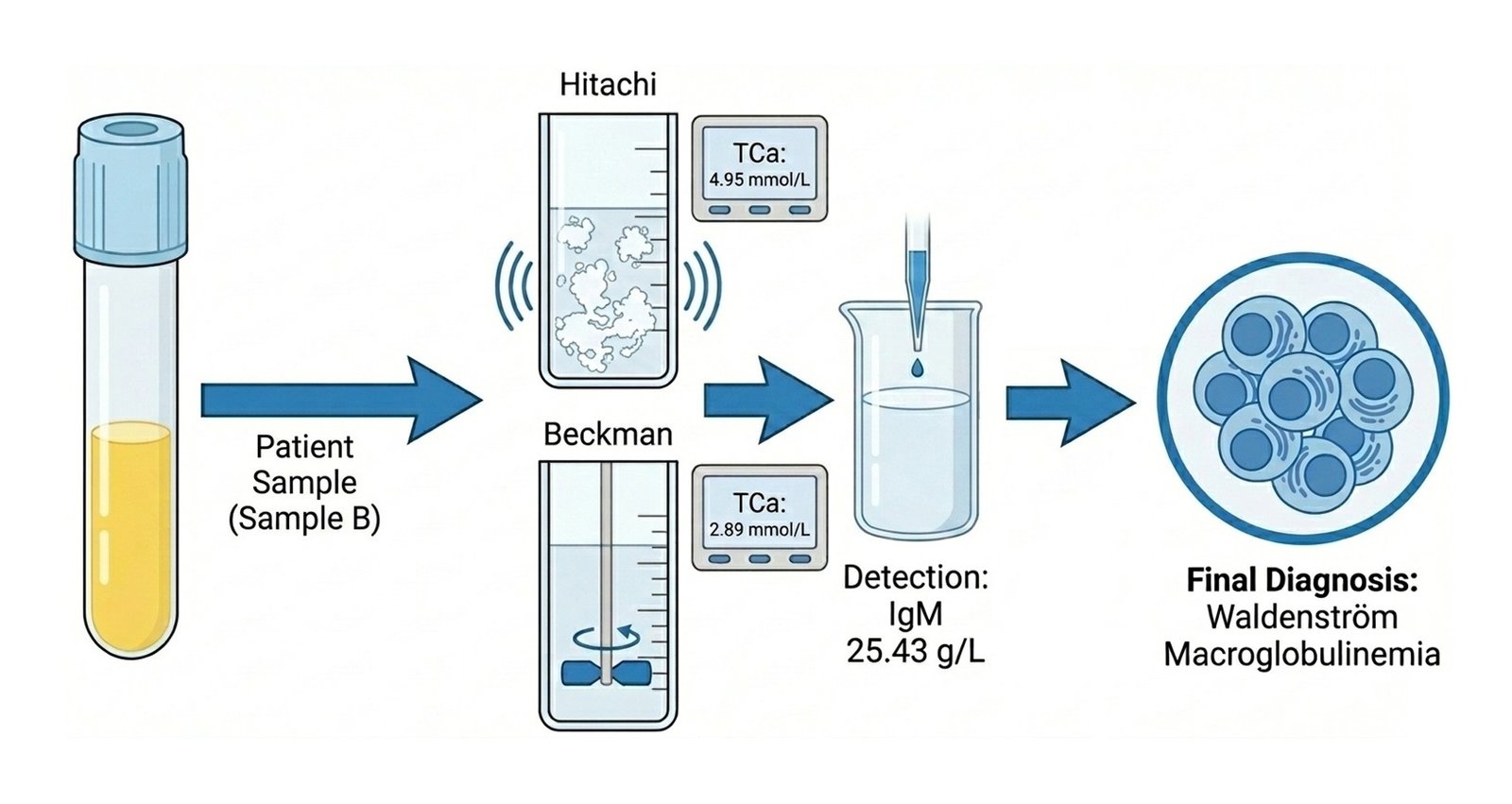

## Highlights

• IgM-κ paraprotein caused marked platform-dependent interference in electrolyte and total calcium measurements

• Total calcium differed by 71% between Hitachi and Beckman despite the same Arsenazo III reagent

• Direct ion-selective electrode analysis excluded true hyponatremia and hypercalcemia

• Platform-specific mixing dynamics determined the extent of paraprotein interference

• Recognition of marked inter-platform discrepancies prompted targeted laboratory investigation, leading to identification of IgM paraprotein and diagnosis of occult Waldenström macroglobulinemia

## Introduction

Waldenström macroglobulinemia (WM) is an indolent B-cell lymphoproliferative disorder characterized by monoclonal IgM paraproteinemia and bone marrow infiltration (*1*-*3*). Its clinical manifestations are diverse and often nonspecific, frequently resulting in delayed diagnosis (*4*-*6*). High concentrations of IgM paraprotein are a well-recognized source of analytical interference and may produce spurious laboratory abnormalities (*7*-*10*). Although pseudohyponatremia and pseudohypercalcemia attributable to IgM have been reported, few studies have systematically evaluated the magnitude of such interference across major analytical platforms.

Here, we describe a patient whose serum sample showed a striking dual pattern of platform-dependent interference on Hitachi and Beckman Coulter platforms. Notably, one platform yielded only clinically subtle abnormalities, which could easily have escaped clinical attention. This case illustrates the importance of correlating laboratory results with clinical findings and pursuing targeted verification when analytical interference is suspected.

## Case report

An 85-year-old man with a 20-year history of hypertension was admitted for low back pain and left thigh pain of 1 year’s duration, with worsening over the preceding week. Lumbar magnetic resonance imaging showed lumbar disc herniation. Vital signs were stable, and physical examination revealed localized tenderness in the left L4–S1 region. On the day of admission, the first venous blood sample was collected from the antecubital vein in the morning in a non-fasting state using a plain vacuum blood collection tube without additives (Zhejiang Gongdong Medical Technology Co., Ltd., China). Initial routine biochemical testing of this sample on a Beckman Coulter AU5811 analyzer (Beckman Coulter, Brea, USA) showed the following results: total calcium (TCa), 2.89 mmol/L (reference interval 2.11-2.52 mmol/L); sodium (Na), 133 mmol/L (137-147 mmol/L); chloride (Cl), 96 mmol/L (99-110 mmol/L); and creatinine, 126 μmol/L (57-111 μmol/L). Although sodium and chloride were only mildly decreased, the unexplained elevation in total calcium was inconsistent with the clinical presentation and was questioned by the clinicians; consequently, they requested a repeat fasting panel, which was collected the following morning and analyzed *via* the laboratory’s routinely available Hitachi LABOSPECT 008α workflow.

The morning after admission, a second fasting venous blood sample was collected from the antecubital vein using the same type of collection tube. Repeated testing on a Hitachi LABOSPECT 008α analyzer (Hitachi High-Technologies Corporation, Tokyo, Japan) revealed a markedly different biochemical profile: TCa 4.95 mmol/L, reaching the threshold for hypercalcemic crisis; Na 125 mmol/L; and Cl 92 mmol/L. Total protein was 78 g/L, albumin was 36 g/L, and globulin was 42 g/L. Despite the extreme abnormalities reported by the Hitachi platform, the patient had no clinical manifestations consistent with severe hypercalcemia, strongly suggesting analytical interference.

Comparison of the initial AU5811 results with the next-day Hitachi results revealed substantial discrepancies in sodium and chloride. Chronologically, the initial biochemical screening (Sample A) was performed on the AU5811 analyzer upon admission. The subsequent LABOSPECT 008α analysis utilized a separate fasting sample (Sample B) collected the following morning. To investigate the profound instrument discordance, Sample B was subjected to multi-platform analysis, including remeasurement on the AU5811 and direct potentiometry on a blood gas analyzer. The AU5811 results displayed in Table 1 represent this re-analysis of Sample B, which exactly matched the index finding from Sample A (yielding a TCa of 2.89 mmol/L). Using the albumin concentration of 36 g/L measured in Sample B, the albumin-corrected total calcium values were calculated as 2.97 mmol/L for the AU5811 remeasurement and 5.03 mmol/L for the LABOSPECT 008α result (*11*). The corrected values remained markedly discrepant, suggesting that albumin adjustment alone was insufficient to account for the observed findings. Importantly, total calcium measured with the same brand and lot of Arsenazo III reagent (Medicalsystem Biotechnology Co., Ltd., Ningbo, China) differed by 2.06 mmol/L, corresponding to a 71.3% inter-platform difference. Given the lack of compatible clinical features, the sample was remeasured on additional platforms and subjected to dilution and confirmatory testing. The results of electrolyte and calcium measurements across analytical platforms, together with dilution and confirmatory testing, are summarized in Table 1.

**Table 1:** Laboratory results of the second-day fasting sample on multiple platforms

**Parameter**	**AU5811 (iISE)**	**LABOSPECT 008α (iISE)**	**ABL90 FLEX (dISE)**	**Reference Interval**
K^+^	3.82	3.73	3.9	3.50–5.30 mmol/L
Na^+^	133	125	138	137–147 mmol/L
Cl^–^	96	92	100	99–110 mmol/L
Ca^2+^	-	-	0.90	1.10–1.35 mmol/L
TCa	2.89	4.95	-	2.11–2.52 mmol/L
TCa (DW 1:1)	1.98	2.00	-	2.11–2.52 mmol/L
TCa (DW 1:3)	2.02	1.96	-	2.11–2.52 mmol/L
TCa (NS 1:1)	2.78	4.56	-	2.11–2.52 mmol/L
TCa (NS 1:3)	2.39	3.32	-	2.11–2.52 mmol/L
TCa (PEG 1:1)	2.00	2.00	-	2.11–2.52 mmol/L
All results shown in this table were obtained from the same fasting serum sample collected on the morning of the second day of admission. Albumin concentration in this sample was 36.1 g/L. The corresponding albumin-corrected total calcium values were 2.97 mmol/L and 5.03 mmol/L for the AU5811 and LABOSPECT 008α results, respectively. Albumin-corrected total calcium was calculated using the formula: corrected TCa (mmol/L) = measured TCa + 0.02 × (40 - albumin (g/L)) (11). Blood gas analysis was performed on an ABL90 FLEX analyzer (Radiometer, Copenhagen, Denmark). For distilled-water dilution and PEG precipitation experiments, measurements were performed on the supernatant obtained after centrifugation. iISE - indirect ion-selective electrode. dISE - direct ion-selective electrode. TCa - total calcium. DW - distilled water. NS - normal saline. PEG - polyethylene glycol 6000, 25%.				

Dilution with distilled water induced immediate opalescence (a positive Sia water test), indicating a high paraprotein concentration susceptible to precipitation under reduced ionic strength (*10*). After 1:1 dilution with distilled water, dilution-adjusted total calcium decreased substantially and approached a level physiologically consistent with the ionized calcium concentration measured by direct ion-selective electrode (dISE, 0.90 mmol/L), confirming analytical interference. With normal saline dilution, paraprotein did not precipitate; however, the interference decreased in a concentration-dependent manner. On the basis of the positive Sia water test and previous reports, macromolecular protein interference affecting sodium, chloride, and total calcium was confirmed (*10*). Polyethylene glycol (PEG 6000, 25%) precipitation was then performed, after which total calcium measured approximately 2.00 mmol/L on the Hitachi platform, supporting the absence of true hypercalcemia demonstrated by the ionized calcium result from the blood gas analyzer.

**Figure 1:**
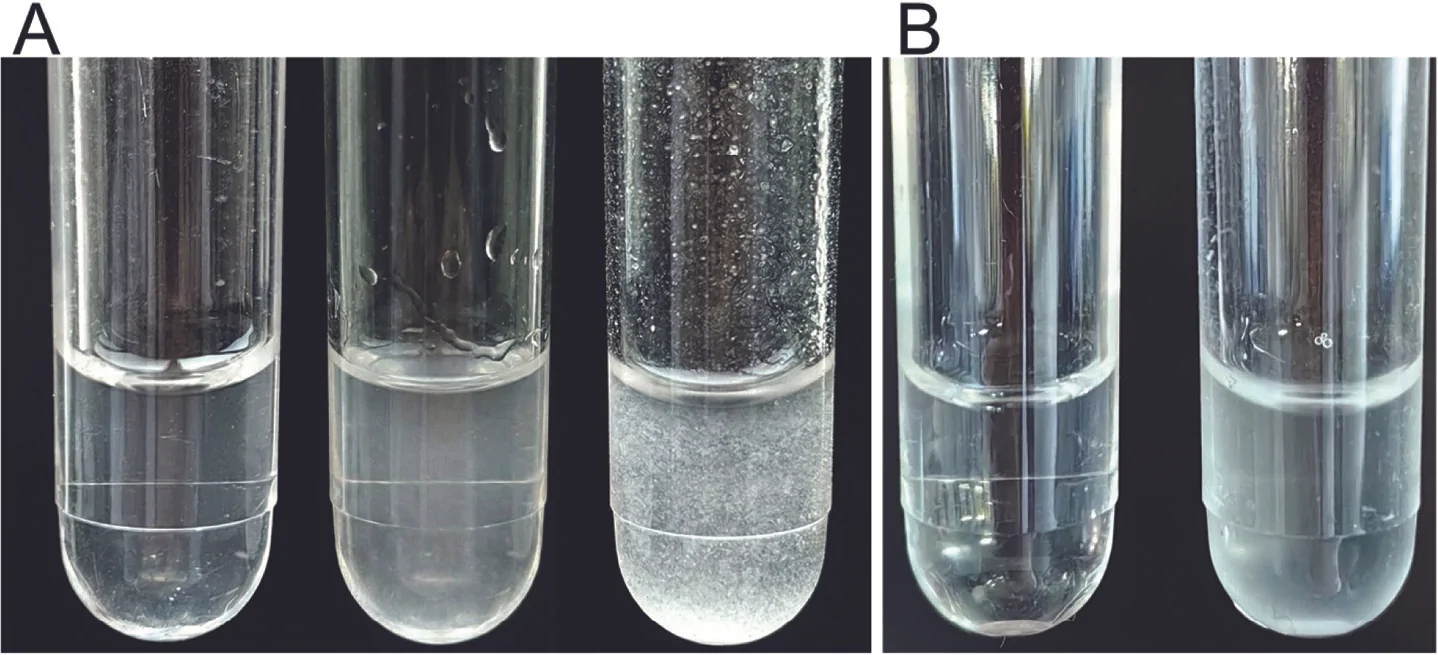
**Visual demonstration of paraprotein-related precipitation during assay mixing. (A)** Mixture of Hitachi ion-selective electrode (ISE) diluent and serum at the routine testing ratio. From left to right: a representative non-paraproteinemic control serum sample mixed with Hitachi diluent without visible precipitation; the patient’s serum mixed with Hitachi diluent approximately 30 s after addition; and the patient’s serum mixed with Hitachi diluent 10 min after addition, showing progressive turbidity. (B) Mixture of Arsenazo III reagent 1 (R1) and serum at the routine testing ratio. The representative non-paraproteinemic control serum sample remained clear, whereas the patient sample developed immediate and marked turbidity on contact with reagent 1.

**Figure 2:**
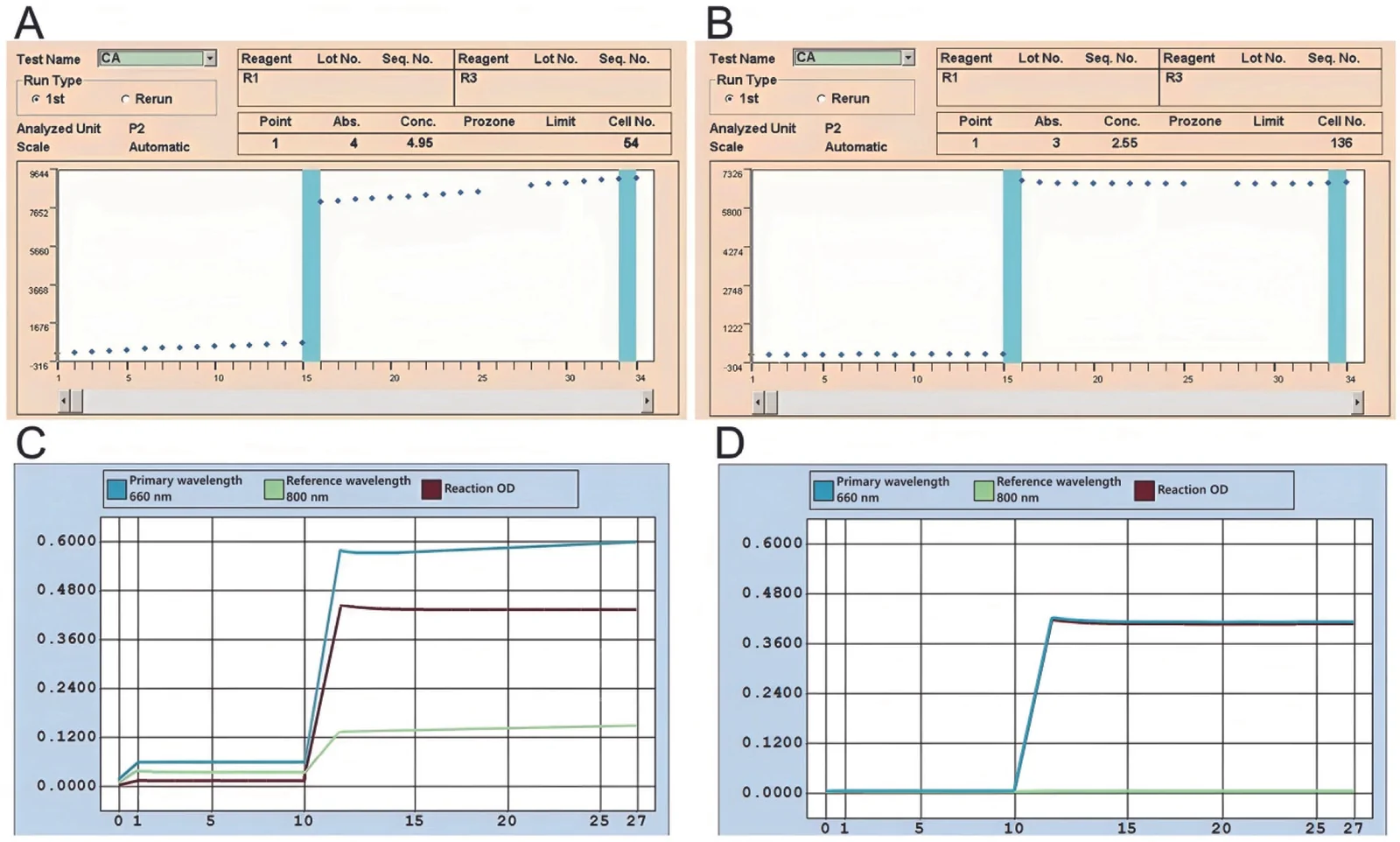
Reaction curve profiles for total calcium (Arsenazo III method) on two analytical platforms. **(A)** Patient sample on the Hitachi platform showing a progressive upward absorbance drift after reagent addition, indicating ongoing paraprotein-induced microprecipitation. **(B)** Representative non-paraproteinemic control serum sample on the Hitachi platform showing a stable reaction plateau. **(C)** Patient sample on the Beckman Coulter platform (reported value, 2.89 mmol/L); despite the slight elevation, the kinetic profile remained relatively stable compared with that shown in panel A. **(D)** Representative non-paraproteinemic control serum sample on the Beckman Coulter platform (reported value, 2.25 mmol/L) showing a normal, stable reaction curve.

Manual simulation experiments further elucidated the mechanism underlying the platform differences. When serum was mixed with the Hitachi ISE diluent, spontaneous flocculent precipitation developed in the patient sample upon static contact (Figure 1A). In contrast, no precipitation was observed with the Beckman Coulter ISE diluent. Similarly, marked turbidity occurred when the patient’s serum contacted Arsenazo III reagent 1 (Figure 1B). Reaction-curve analysis demonstrated pronounced platform-dependent differences (Figure 2). On the Hitachi platform, absorbance showed a continuous upward drift, reflecting persistent optical scattering from suspended precipitates generated by ultrasonic mixing. In contrast, the Beckman Coulter AU platform showed relatively stable kinetics. Nevertheless, the final reported total calcium concentrations on the Beckman Coulter AU platform were 2.89 mmol/L for the patient sample and 2.25 mmol/L for the representative non-paraproteinemic control serum sample.

Based on these findings, immunoglobulin testing was subsequently requested and revealed markedly elevated IgM (25.43 g/L), identifying IgM paraprotein as the principal cause of the observed analytical interference. A comprehensive hematological evaluation was therefore initiated. Bone marrow examination revealed lymphoplasmacytic infiltration, and bone marrow cytology was concordant with the histological findings. Immunophenotypic analysis by flow cytometry demonstrated a clonal population of kappa light chain-restricted B cells with a phenotype consistent with lymphoplasmacytic lymphoma. Molecular testing further demonstrated MYD88 L265P positivity, whereas no CXCR4 mutation was detected. Taken together with the markedly elevated serum IgM concentration, these findings established the diagnosis of WM.

Given the presence of renal dysfunction and light-chain involvement, the patient subsequently received targeted therapy with the Bruton tyrosine kinase inhibitor ibrutinib (420 mg once daily). After treatment initiation, he showed a favorable clinical and laboratory response. After 3 months of treatment, he reported marked relief of back and lower limb pain. At 6 months of follow-up, hematological parameters indicated a substantial response: serum IgM had decreased from 25.43 g/L to 2.08 g/L, and the M-protein fraction had declined to 2.7%. Renal function and metabolic parameters improved concurrently, with serum creatinine normalizing to 76 μmol/L and estimated glomerular filtration rate increasing to 92.7 mL/min/1.73 m^2^. Total calcium returned to the normal range and stabilized at approximately 2.35 mmol/L. These findings indicate that early laboratory recognition of analytical interference not only prevented misinterpretation of biochemical results, but also facilitated timely diagnosis and appropriate management of the underlying hematological disorder. Unfortunately, the patient later died of COVID-19-related complications. Written informed consent for the publication of this case report and the accompanying images was obtained from the patient’s next of kin.

## Discussion

A particularly important feature of this case is that total calcium on the Beckman Coulter AU5811 was only mildly elevated at 2.89 mmol/L. Such values often receive limited clinical attention and would rarely be attributed to severe paraprotein-related interference. In contrast, the spurious “hypercalcemic crisis” reported by the Hitachi platform was not clinically plausible and therefore triggered verification for analytical interference. This finding emphasizes that any discrepancy between laboratory metrics and clinical presentation demands immediate investigation. The 71% inter-platform discrepancy prompted further investigation that ultimately led to diagnosis.

With respect to the electrolyte discrepancies, indirect ion-selective electrode results differed markedly between platforms. On the Beckman Coulter AU5811, macroglobulins occupy plasma volume during dilution, causing underestimation of sodium and chloride through the electrolyte exclusion effect (*12*-*15*). On the Hitachi platform, this interference was further amplified by precipitation and reduced water availability, resulting in more pronounced pseudohyponatremia.

The extreme discrepancy between the Hitachi and Beckman Coulter platforms, together with similar observations reported between Hitachi and Siemens systems, supports the existence of a platform-dependent interference gradient for Arsenazo III methods (*16*). Crucially, standard albumin correction did not materially alter this discrepancy, indicating that albumin adjustment alone could not explain the observed findings and that the discrepancy was unlikely to reflect altered protein-binding physiology alone. Instead, differences in mixing methodology, particularly the high-energy ultrasonic mixing used by the Hitachi platform, likely determine the extent to which precipitates remain suspended within the optical path, thereby substantially influencing absorbance and the final reported calcium concentration (*17*, *18*). Thus, despite use of the same Arsenazo III principle, platform-specific mixing and reaction conditions appear to critically modulate the magnitude of paraprotein-induced optical interference.

This case extends previous observations by showing that unexplained analytical abnormalities may delay recognition of underlying disease if analytical interference is not considered. Multiple reports have described combined pseudohyponatremia and pseudohypercalcemia caused by monoclonal paraproteins, particularly IgM (*19*-*22*). Paraproteins may also interfere with numerous other assays, including enzymes, creatinine, hemolysis indices, and bicarbonate (*23*-*27*).

## Implications for laboratory practice

The practical lesson from this case is not that all samples should be routinely tested on multiple chemistry platforms. Rather, when laboratory results are unexplained or inconsistent with clinical expectations, even mild abnormalities should prompt consideration of analytical interference. To avoid misdiagnosis in similar settings, identifying the cause of major discrepancies between analytical platforms is essential. In addition, when mild abnormalities are unexplained, inspection of reaction curves may help identify interference that might otherwise be overlooked.

When the clinical presentation is discordant with biochemical findings, for example in an asymptomatic patient with an apparent “hypercalcemic crisis”, visual inspection of the reaction curve and the sample itself, including the Sia water test, may be informative. Furthermore, standard automated flags on a routine chemistry panel, such as an elevated globulin fraction and an inverted albumin-to-globulin (A/G) ratio, serve as critical, universally accessible clues signaling the potential presence of high-concentration monoclonal immunoglobulins. Hemolysis, icterus, and lipemia indices may also provide useful clues to abnormal sample properties. Simple mixing tests with water or saline can reveal precipitation in selected high-paraprotein samples, although precipitation may depend on ionic strength and reagent composition. Use of direct ISE or dilution-based approaches provides an important verification step when high protein concentrations are suspected. For laboratories operating a single routine chemistry platform, a blood gas analyzer equipped with direct ISE serves as a practical alternative for electrolyte verification. If such testing is not available locally, external verification, dilution studies, PEG precipitation, and close clinical-laboratory communication should be considered.

Ultimately, proactive communication between the laboratory and clinicians plays a pivotal role in the diagnosis of occult hematological disorders. Laboratory professionals should interpret results from a clinical perspective and actively discuss unexpected or clinically inconsistent findings with clinicians to seek a reasonable explanation.

## Data Availability

All data generated and analyzed in the presented study are included in this published article.
